# 2,4,6-Tri­nitro­phenyl 3-bromo­benzoate

**DOI:** 10.1107/S1600536814010952

**Published:** 2014-05-21

**Authors:** Rodolfo Moreno-Fuquen, Fabricio Mosquera, Javier Ellena, Juan C. Tenorio

**Affiliations:** a Departamento de Química - Facultad de Ciencias Naturales y Exactas, Universidad del Valle, Apartado 25360, Santiago de Cali, Colombia; b Instituto de Física de São Carlos, IFSC, Universidade de São Paulo, USP, São Carlos, SP, Brazil

## Abstract

In the title picryl-substituted ester, C_13_H_6_BrN_3_O_8_, the mean plane of the central ester C–O–C(=O)–C fragment (r.m.s. deviation= 0.0186 Å) is rotated by 84.73 (7)° and 19.92 (12)° to the picryl and phenyl rings, respectively. In the crystal, the mol­ecules are linked by C—H⋯O inter­actions, forming centrosymmetric dimers enclosing *R* ^2^_2_(10) and *R* ^2^_2_(22) ring motifs along [001] and further helical chains of dimers enclosing *R* ^2^_2_(10) ring motifs along [010].

## Related literature   

For related structures, including isostructural 2,4,6-tri­nitro­phenyl 3-chloro­benzoate, see: Moreno-Fuquen *et al.* (2013*a* ▶,*b* ▶,*c* ▶). For a detailed study of the central ester moiety, see: Moreno-Fuquen *et al.* (2012 ▶). For hydrogen bonding, see: Nardelli (1995 ▶) and for hydrogen-bond graph-set motifs, see: Etter (1990 ▶).
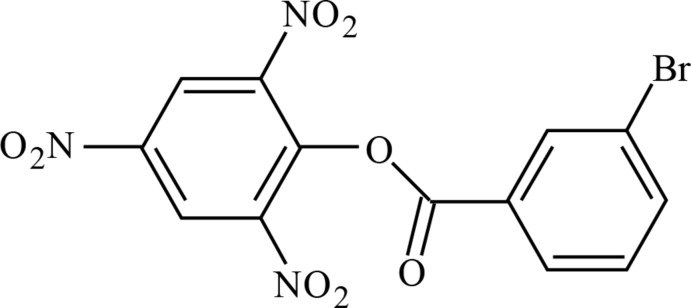


## Experimental   

### 

#### Crystal data  

C_13_H_6_BrN_3_O_8_
*M* *_r_* = 412.12
Monoclinic, 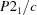

*a* = 11.2925 (4) Å
*b* = 9.5672 (3) Å
*c* = 14.0560 (6) Å
β = 94.625 (2)°
*V* = 1513.63 (10) Å^3^
*Z* = 4
Mo *K*α radiation
μ = 2.77 mm^−1^
*T* = 295 K
0.27 × 0.24 × 0.13 mm

#### Data collection  

Nonius KappaCCD diffractometer
Absorption correction: multi-scan (*SADABS*; Sheldrick, 2004 ▶) *T*_min_ = 0.486, *T*_max_ = 0.601
5971 measured reflections
3082 independent reflections
2572 reflections with *I* > 2σ(*I*)
*R*_int_ = 0.021

#### Refinement  

*R*[*F* ^2^ > 2σ(*F* ^2^)] = 0.031
*wR*(*F* ^2^) = 0.081
*S* = 1.03
3082 reflections
227 parameters
H-atom parameters constrained
Δρ_max_ = 0.42 e Å^−3^
Δρ_min_ = −0.50 e Å^−3^

### 

Data collection: *COLLECT* (Nonius, 2000 ▶); cell refinement: *HKL* *SCALEPACK* (Otwinowski & Minor, 1997 ▶); data reduction: *HKL* *DENZO* (Otwinowski & Minor, 1997 ▶) and *SCALEPACK*; program(s) used to solve structure: *SHELXS97* (Sheldrick, 2008 ▶); program(s) used to refine structure: *SHELXL97* (Sheldrick, 2008 ▶); molecular graphics: *ORTEP-3 for Windows* (Farrugia, 2012 ▶) and *Mercury* (Macrae *et al.*, 2006 ▶); software used to prepare material for publication: *WinGX* (Farrugia, 2012 ▶).

## Supplementary Material

Crystal structure: contains datablock(s) I, global. DOI: 10.1107/S1600536814010952/fk2082sup1.cif

Structure factors: contains datablock(s) I. DOI: 10.1107/S1600536814010952/fk2082Isup2.hkl

Supporting information file. DOI: 10.1107/S1600536814010952/fk2082Isup3.cml

CCDC reference: 1002544

Additional supporting information:  crystallographic information; 3D view; checkCIF report

## Figures and Tables

**Table 1:** Hydrogen-bond geometry (Å, °)

*D*—H⋯*A*	*D*—H	H⋯*A*	*D*⋯*A*	*D*—H⋯*A*
C5—H5⋯O4^i^	0.93	2.51	3.140 (3)	125
C11—H11⋯O6^ii^	0.93	2.46	3.282 (3)	148
C13—H13⋯O3^iii^	0.93	2.40	3.314 (3)	166
C3—H3⋯O8^iv^	0.93	2.46	3.391 (3)	175

Symmetry codes: (i) 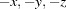; (ii) 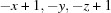; (iii) 
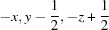
; (iv) 
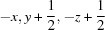
.

**Fig. 1.**
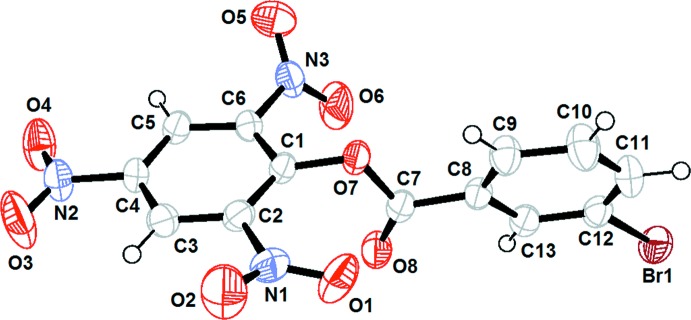
Molecular structure of the title compound with displacement ellipsoids drawn at the 50% probability level. H atoms are shown as spheres of arbitrary radius.

**Fig. 2.**
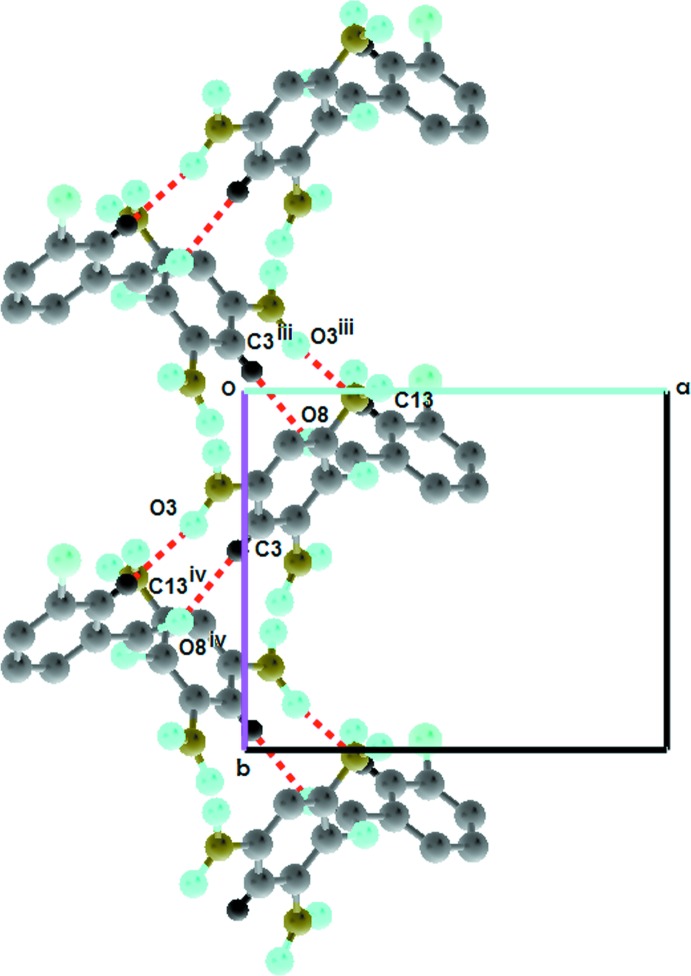
Part of the crystal structure showing the formation of helical rings running along [010]. H-atoms not involved are omitted. Symmetry code: (i) -x, y-1/2, -z+1/2; (ii) -x, y+1/2, -z+1/2.

**Fig. 3.**
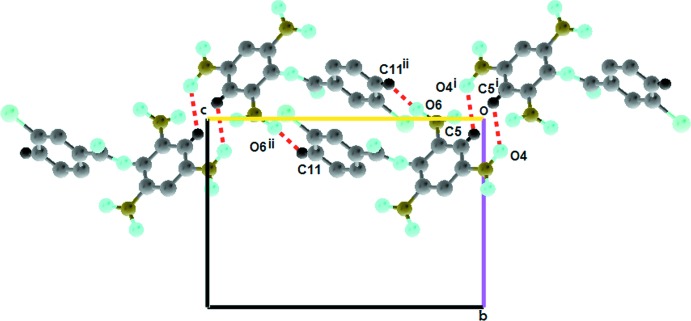
Part of the crystal structure showing the formation of helical rings running along [001]. H-atoms not involved are omitted. Symmetry code: (iii) -x,-y,-z; (iv) -x+1,-y,-z+1.

## supplementary crystallographic information

### 1. Comment

The crystal structure determination of 2,4,6-trinitrophenyl 3-bromobenzoate (I), a picryl substituted-ester, is presented as part of an extensive study carried out in our research group about this type of compounds. Recently, we have published similar structures: 2,4,6-trinitrophenyl 4-bromobenzoate (Moreno-Fuquen, 2013*a*) and 2,4,6-trinitrophenyl 2-furancarboxylate (Moreno-Fuquen *et al.*, 2013*b*). The molecular structure of (I) is shown in Fig. 1. The picryl and phenyl rings form angles of 84.73 (7)° and 19.92 (12)° respectively with the ester fragment. The nitro groups form dihedral angles with the adjacent benzene ring of 19.96 (14)°, 4.07 (20)° and 55.79 (9)° for O1–N1–O2, O3–N2–O4 and O5–N3–O6, respectively. The structure is essentially isotypic with the known chloro compound (Moreno-Fuquen *et al.*, 2013*c*), thus the packing shows the same structural relationship as discussed for the chlorobenzoate. The molecules are packed through weak C–H···O interactions forming a three dimensional network (see Table 1, Nardelli, 1995). Considering the strongest C—H···O contacts, there are two mainly growth directions: one along b and the other one along c which are explained in terms of the substructures shown in Fig. 2 and 3 respectively. In the first one, C13-H13···O3 and C3-H3···O8 contacts, which reinforced each other, allow the molecules to propagate forming one-dimensional helical chains, along [010]. The C13 atom of the phenyl ring at (x,y,z) acts as a hydrogen-bond donor to O3 atom of the nitro group at (-x, y-1/2, -z+1/2) and C3 atom of the picryl ring at (x,y,z) acts as hydrogen bond donor to carbonyl O8 atom at (-x, y+1/2, -z+1/2). The combination of these two contacts generate edge-fused rings R^2^_2_(10) (Etter, 1990), (see Fig.2). This molecular synthon seems to be a common feature along picryl substituted-esters (Moreno-Fuquen *et al.*, 2012). In the second substructure (Fig. 3), the additional weak C5-H5···O4 and C11-H11···O6 interactions, both forming dimers by an inversion centre within the structure with R^2^_2_(10) and R^2^_2_(22) edge-fused rings, allow the molecules to grow alternating dimers along [001]. The C5 atom of the picryl ring at (x,y,z) acts as hydrogen bond donor to O4 of the nitro group at (-x,-y,-z) and C11 atom of the phenyl ring at (x,y,z) acts as hydrogen bond donor to O6 atom of the nitro group at (-x+1,-y,-z+1).

### 2. Experimental

The reagents and solvents for the synthesis were obtained from the Aldrich Chemical Co., and were used without additional purification. The title molecule was obtained through a two-step reaction. First, 3-bromobenzoic acid (0.100 g, 0.243 mmol) was refluxed in excess of thionyl chloride (10 ml) for an hour. Then, the thionyl chloride was distilled off under reduced pressure to purify the 3-bromobenzoyl chloride obtained as a pale-yellow translucent liquid. The same reaction flask was rearranged and a solution of picric acid (0.060 g, 0.243 mmol) in acetonitrile was dropped inside it with constant stirring. The reaction mixture was taken to reflux for about an hour. A pale-yellow solid was obtained after leaving the solvent to evaporate. This was washed with distilled water and cold methanol to eliminate impurities. Pale-yellow crystals of good quality [92% yield, m.p. 396 (1) K] and suitable for single-crystal X-ray diffraction were grown in acetonitrile.

### 3. Refinement

All H-atoms were positioned at geometrically idealized positions, C—H= 0.93Å, and they were refined using a riding model approximation with *U*_iso_(H) constrained to 1.2 times *U*_eq_ of the respective parent atom.

### Crystal data

**Table:**

C_13_H_6_BrN_3_O_8_	*F*(000) = 816
*M**_r_* = 412.12	*D*_x_ = 1.808 Mg m^−^^3^
Monoclinic, *P*2_1_/*c*	Melting point: 396(1) K
Hall symbol: -P 2ybc	Mo *K*α radiation, λ = 0.71073 Å
*a* = 11.2925 (4) Å	Cell parameters from 5804 reflections
*b* = 9.5672 (3) Å	θ = 3.1–26.4°
*c* = 14.0560 (6) Å	µ = 2.77 mm^−^^1^
β = 94.625 (2)°	*T* = 295 K
*V* = 1513.63 (10) Å^3^	Block, pale-yellow
*Z* = 4	0.27 × 0.24 × 0.13 mm

### Data collection

**Table:**

Nonius KappaCCD diffractometer	3082 independent reflections
Radiation source: fine-focus sealed tube	2572 reflections with *I* > 2σ(*I*)
Graphite monochromator	*R*_int_ = 0.021
CCD rotation images, thick slices scans	θ_max_ = 26.4°, θ_min_ = 3.1°
Absorption correction: multi-scan (*SADABS*; Sheldrick, 2004)	*h* = −14→14
*T*_min_ = 0.486, *T*_max_ = 0.601	*k* = −11→11
5971 measured reflections	*l* = −17→17

### Refinement

**Table:**

Refinement on *F*^2^	Secondary atom site location: difference Fourier map
Least-squares matrix: full	Hydrogen site location: inferred from neighbouring sites
*R*[*F*^2^ > 2σ(*F*^2^)] = 0.031	H-atom parameters constrained
*wR*(*F*^2^) = 0.081	*w* = 1/[σ^2^(*F*_o_^2^) + (0.0403*P*)^2^ + 0.7085*P*] where *P* = (*F*_o_^2^ + 2*F*_c_^2^)/3
*S* = 1.03	(Δ/σ)_max_ < 0.001
3082 reflections	Δρ_max_ = 0.42 e Å^−^^3^
227 parameters	Δρ_min_ = −0.50 e Å^−^^3^
0 restraints	Extinction correction: *SHELXL*, Fc^*^=kFc[1+0.001xFc^2^λ^3^/sin(2θ)]^-1/4^
Primary atom site location: structure-invariant direct methods	Extinction coefficient: 0.0122 (8)

### Special details

**Table:**

Geometry. All esds (except the esd in the dihedral angle between two l.s. planes) are estimated using the full covariance matrix. The cell esds are taken into account individually in the estimation of esds in distances, angles and torsion angles; correlations between esds in cell parameters are only used when they are defined by crystal symmetry. An approximate (isotropic) treatment of cell esds is used for estimating esds involving l.s. planes.
Refinement. Refinement of *F*^2^ against ALL reflections. The weighted *R*-factor *wR* and goodness of fit *S* are based on *F*^2^, conventional *R*-factors *R* are based on *F*, with *F* set to zero for negative *F*^2^. The threshold expression of *F*^2^ > σ(*F*^2^) is used only for calculating *R*-factors(gt) *etc*. and is not relevant to the choice of reflections for refinement. *R*-factors based on *F*^2^ are statistically about twice as large as those based on *F*, and *R*- factors based on ALL data will be even larger.

### Fractional atomic coordinates and isotropic or equivalent isotropic displacement parameters (Å^2^)

**Table:**

	*x*	*y*	*z*	*U*_iso_*/*U*_eq_	
Br1	0.42777 (2)	−0.02749 (3)	0.715747 (17)	0.05304 (13)	
O1	0.1739 (2)	0.4619 (2)	0.36624 (13)	0.0651 (5)	
O2	0.0830 (2)	0.5890 (2)	0.25761 (16)	0.0814 (7)	
O3	−0.1211 (2)	0.3737 (3)	−0.01520 (17)	0.0930 (9)	
O4	−0.06765 (18)	0.1728 (2)	−0.06105 (14)	0.0635 (5)	
O5	0.3193 (2)	−0.0080 (2)	0.10541 (17)	0.0731 (6)	
O6	0.2583 (2)	−0.0484 (2)	0.24323 (15)	0.0713 (6)	
O7	0.28431 (12)	0.23650 (17)	0.30276 (10)	0.0385 (4)	
O8	0.15437 (13)	0.14296 (19)	0.39805 (11)	0.0453 (4)	
N1	0.12577 (19)	0.4786 (2)	0.28662 (15)	0.0457 (5)	
N2	−0.06070 (18)	0.2704 (2)	−0.00614 (15)	0.0489 (5)	
N3	0.26169 (17)	0.0199 (2)	0.17035 (15)	0.0445 (5)	
C1	0.19417 (17)	0.2459 (2)	0.23105 (14)	0.0335 (5)	
C2	0.11868 (19)	0.3606 (2)	0.21900 (15)	0.0360 (5)	
C3	0.03478 (19)	0.3701 (2)	0.14191 (15)	0.0385 (5)	
H3	−0.0167	0.4460	0.1351	0.046*	
C4	0.02977 (18)	0.2642 (2)	0.07569 (14)	0.0356 (5)	
C5	0.10472 (18)	0.1513 (2)	0.08195 (15)	0.0364 (5)	
H5	0.1018	0.0829	0.0348	0.044*	
C6	0.18467 (18)	0.1439 (2)	0.16128 (14)	0.0342 (5)	
C7	0.25235 (18)	0.1838 (2)	0.38886 (14)	0.0355 (5)	
C8	0.35354 (18)	0.1859 (2)	0.46246 (14)	0.0350 (5)	
C9	0.4515 (2)	0.2719 (3)	0.45653 (16)	0.0435 (5)	
H9	0.4574	0.3296	0.4039	0.052*	
C10	0.5406 (2)	0.2703 (3)	0.53043 (18)	0.0531 (6)	
H10	0.6060	0.3288	0.5276	0.064*	
C11	0.5337 (2)	0.1834 (3)	0.60789 (17)	0.0482 (6)	
H11	0.5939	0.1829	0.6571	0.058*	
C12	0.43635 (19)	0.0970 (2)	0.61152 (15)	0.0383 (5)	
C13	0.34547 (18)	0.0974 (2)	0.54011 (15)	0.0368 (5)	
H13	0.2799	0.0394	0.5437	0.044*	

### Atomic displacement parameters (Å^2^)

**Table:**

	*U*^11^	*U*^22^	*U*^33^	*U*^12^	*U*^13^	*U*^23^
Br1	0.06119 (19)	0.0611 (2)	0.03652 (16)	0.01162 (12)	0.00239 (11)	0.01329 (11)
O1	0.0973 (15)	0.0632 (13)	0.0334 (10)	−0.0016 (11)	−0.0035 (9)	−0.0101 (9)
O2	0.1105 (18)	0.0569 (14)	0.0728 (14)	0.0343 (13)	−0.0170 (12)	−0.0218 (11)
O3	0.1003 (16)	0.0825 (16)	0.0870 (16)	0.0552 (14)	−0.0486 (13)	−0.0217 (13)
O4	0.0767 (12)	0.0491 (11)	0.0588 (12)	−0.0011 (10)	−0.0308 (10)	−0.0065 (9)
O5	0.0713 (13)	0.0773 (15)	0.0722 (15)	0.0310 (11)	0.0148 (11)	−0.0117 (11)
O6	0.0920 (15)	0.0594 (13)	0.0594 (13)	0.0284 (11)	−0.0131 (11)	0.0154 (10)
O7	0.0352 (7)	0.0526 (10)	0.0269 (7)	−0.0028 (7)	−0.0020 (6)	0.0040 (7)
O8	0.0388 (8)	0.0594 (11)	0.0369 (8)	−0.0083 (8)	−0.0021 (6)	0.0078 (8)
N1	0.0504 (11)	0.0471 (13)	0.0404 (11)	0.0032 (10)	0.0086 (9)	−0.0076 (9)
N2	0.0510 (12)	0.0476 (13)	0.0453 (11)	0.0063 (10)	−0.0137 (9)	0.0030 (10)
N3	0.0431 (11)	0.0434 (12)	0.0447 (12)	0.0097 (9)	−0.0103 (9)	−0.0057 (10)
C1	0.0333 (10)	0.0419 (12)	0.0249 (9)	−0.0002 (9)	0.0009 (8)	0.0042 (9)
C2	0.0408 (11)	0.0375 (12)	0.0302 (10)	0.0016 (10)	0.0052 (9)	−0.0017 (9)
C3	0.0404 (11)	0.0371 (12)	0.0379 (12)	0.0052 (10)	0.0034 (9)	0.0038 (10)
C4	0.0385 (11)	0.0381 (12)	0.0289 (10)	0.0024 (10)	−0.0044 (8)	0.0055 (9)
C5	0.0430 (11)	0.0369 (12)	0.0286 (10)	0.0014 (10)	−0.0009 (9)	0.0004 (9)
C6	0.0354 (10)	0.0360 (12)	0.0309 (10)	0.0057 (9)	0.0004 (8)	0.0040 (9)
C7	0.0394 (11)	0.0384 (12)	0.0283 (10)	0.0006 (10)	0.0000 (8)	0.0011 (9)
C8	0.0365 (10)	0.0388 (12)	0.0289 (10)	0.0022 (10)	−0.0013 (8)	−0.0013 (9)
C9	0.0451 (12)	0.0461 (14)	0.0382 (12)	−0.0038 (11)	−0.0030 (10)	0.0090 (10)
C10	0.0452 (13)	0.0570 (16)	0.0545 (15)	−0.0135 (12)	−0.0116 (11)	0.0084 (13)
C11	0.0445 (12)	0.0549 (15)	0.0423 (13)	0.0012 (12)	−0.0136 (10)	0.0027 (12)
C12	0.0445 (12)	0.0401 (13)	0.0300 (10)	0.0103 (10)	0.0008 (9)	0.0011 (9)
C13	0.0378 (11)	0.0392 (13)	0.0332 (11)	−0.0003 (10)	0.0016 (9)	−0.0002 (9)

### Geometric parameters (Å, º)

**Table:**

Br1—C12	1.897 (2)	C3—C4	1.374 (3)
O1—N1	1.215 (3)	C3—H3	0.9300
O2—N1	1.218 (3)	C4—C5	1.370 (3)
O3—N2	1.201 (3)	C5—C6	1.379 (3)
O4—N2	1.210 (3)	C5—H5	0.9300
O5—N3	1.193 (3)	C7—C8	1.478 (3)
O6—N3	1.218 (3)	C8—C9	1.387 (3)
O7—C1	1.376 (2)	C8—C13	1.391 (3)
O7—C7	1.386 (2)	C9—C10	1.387 (3)
O8—C7	1.190 (2)	C9—H9	0.9300
N1—C2	1.474 (3)	C10—C11	1.377 (3)
N2—C4	1.477 (3)	C10—H10	0.9300
N3—C6	1.470 (3)	C11—C12	1.379 (3)
C1—C6	1.382 (3)	C11—H11	0.9300
C1—C2	1.392 (3)	C12—C13	1.376 (3)
C2—C3	1.384 (3)	C13—H13	0.9300
			
C1—O7—C7	115.73 (16)	C5—C6—C1	123.1 (2)
O1—N1—O2	123.9 (2)	C5—C6—N3	117.15 (19)
O1—N1—C2	119.4 (2)	C1—C6—N3	119.79 (18)
O2—N1—C2	116.7 (2)	O8—C7—O7	121.61 (18)
O3—N2—O4	124.0 (2)	O8—C7—C8	126.98 (19)
O3—N2—C4	117.8 (2)	O7—C7—C8	111.41 (17)
O4—N2—C4	118.1 (2)	C9—C8—C13	120.8 (2)
O5—N3—O6	125.4 (2)	C9—C8—C7	122.8 (2)
O5—N3—C6	118.0 (2)	C13—C8—C7	116.42 (19)
O6—N3—C6	116.5 (2)	C8—C9—C10	118.9 (2)
O7—C1—C6	118.96 (19)	C8—C9—H9	120.6
O7—C1—C2	123.38 (19)	C10—C9—H9	120.6
C6—C1—C2	117.32 (18)	C11—C10—C9	121.0 (2)
C3—C2—C1	121.4 (2)	C11—C10—H10	119.5
C3—C2—N1	116.9 (2)	C9—C10—H10	119.5
C1—C2—N1	121.65 (19)	C10—C11—C12	119.1 (2)
C4—C3—C2	118.0 (2)	C10—C11—H11	120.4
C4—C3—H3	121.0	C12—C11—H11	120.4
C2—C3—H3	121.0	C13—C12—C11	121.4 (2)
C5—C4—C3	123.1 (2)	C13—C12—Br1	118.90 (17)
C5—C4—N2	117.8 (2)	C11—C12—Br1	119.68 (16)
C3—C4—N2	119.1 (2)	C12—C13—C8	118.8 (2)
C4—C5—C6	117.0 (2)	C12—C13—H13	120.6
C4—C5—H5	121.5	C8—C13—H13	120.6
C6—C5—H5	121.5		
			
C7—O7—C1—C6	−100.4 (2)	C2—C1—C6—C5	−0.1 (3)
C7—O7—C1—C2	86.5 (3)	O7—C1—C6—N3	6.8 (3)
O7—C1—C2—C3	175.27 (19)	C2—C1—C6—N3	−179.71 (19)
C6—C1—C2—C3	2.0 (3)	O5—N3—C6—C5	54.2 (3)
O7—C1—C2—N1	−3.6 (3)	O6—N3—C6—C5	−123.6 (2)
C6—C1—C2—N1	−176.81 (19)	O5—N3—C6—C1	−126.1 (2)
O1—N1—C2—C3	161.2 (2)	O6—N3—C6—C1	56.0 (3)
O2—N1—C2—C3	−19.6 (3)	C1—O7—C7—O8	3.6 (3)
O1—N1—C2—C1	−19.9 (3)	C1—O7—C7—C8	−176.60 (18)
O2—N1—C2—C1	159.3 (2)	O8—C7—C8—C9	−159.4 (2)
C1—C2—C3—C4	−1.6 (3)	O7—C7—C8—C9	20.8 (3)
N1—C2—C3—C4	177.32 (19)	O8—C7—C8—C13	19.8 (3)
C2—C3—C4—C5	−0.9 (3)	O7—C7—C8—C13	−159.95 (19)
C2—C3—C4—N2	178.70 (19)	C13—C8—C9—C10	−1.3 (4)
O3—N2—C4—C5	−175.1 (3)	C7—C8—C9—C10	177.9 (2)
O4—N2—C4—C5	3.3 (3)	C8—C9—C10—C11	1.1 (4)
O3—N2—C4—C3	5.3 (4)	C9—C10—C11—C12	−0.1 (4)
O4—N2—C4—C3	−176.3 (2)	C10—C11—C12—C13	−0.9 (4)
C3—C4—C5—C6	2.7 (3)	C10—C11—C12—Br1	178.1 (2)
N2—C4—C5—C6	−176.87 (19)	C11—C12—C13—C8	0.7 (3)
C4—C5—C6—C1	−2.2 (3)	Br1—C12—C13—C8	−178.21 (16)
C4—C5—C6—N3	177.4 (2)	C9—C8—C13—C12	0.4 (3)
O7—C1—C6—C5	−173.62 (19)	C7—C8—C13—C12	−178.9 (2)

### Hydrogen-bond geometry (Å, º)

**Table:**

*D*—H···*A*	*D*—H	H···*A*	*D*···*A*	*D*—H···*A*
C5—H5···O4^i^	0.93	2.51	3.140 (3)	125
C11—H11···O6^ii^	0.93	2.46	3.282 (3)	148
C13—H13···O3^iii^	0.93	2.40	3.314 (3)	166
C3—H3···O8^iv^	0.93	2.46	3.391 (3)	175

Symmetry codes: (i) −*x*, −*y*, −*z*; (ii) −*x*+1, −*y*, −*z*+1; (iii) −*x*, *y*−1/2, −*z*+1/2; (iv) −*x*, *y*+1/2, −*z*+1/2.
